# Right-Sided Aortic Arch With Isolated Left Subclavian Artery, Subclavian Steal Syndrome, and Right Bronchial Compression: A Case Report

**DOI:** 10.7759/cureus.59961

**Published:** 2024-05-09

**Authors:** Faisal Mulla, Anusha Shree Thaneeru, Jeevika M Ujjappa

**Affiliations:** 1 Radiodiagnosis, Jaya Jagadguru Murugharajendra (JJM) Medical College, Davangere, IND

**Keywords:** subclavian steal syndrome, aortic arch anomalies, tracheal compression, isolated left subclavian artery, right aortic arch

## Abstract

A right-sided aortic arch with an isolated left subclavian artery represents a rare anatomical variant, posing diagnostic challenges and clinical complexities. Here, we present a case of a 14-year-old male presenting with respiratory symptoms, unveiling a right-sided aortic arch with an isolated left subclavian artery. Through detailed clinical evaluation, radiographic imaging, and diagnostic modalities including chest radiography, computed tomography angiography, ultrasound, and time-of-flight magnetic resonance angiography, the anatomical features and associated complications were delineated. The discussion encompasses embryological underpinnings, clinical manifestations, and therapeutic considerations, shedding light on the rarity and clinical implications of this anomaly.

## Introduction

A right-sided aortic arch is an anatomical variation of the aortic arch characterized by its course to the right of the trachea, with its incidence reported to be between 0.05% and 0.1% [1,2]. Three types of right-sided aortic arches have been described in the literature [3-5], each of which is associated with a separate set of complications. We describe a case of right-sided aortic arch with an isolated left subclavian artery, which has been described as the third type and is the rarest among the three, with its incidence being 0.8% [2], and is associated with subclavian steal syndrome.

## Case presentation

A 14-year-old male child presented with episodic cough for the past four days, spasmodic in nature, which was associated with breathlessness. These episodes lasted for 2-4 minutes. The child gave no history of similar episodes in the past. On general physical examination, the pulse rate was normal, and the blood pressure, which was measured in the right arm, was 110/74 mmHg. On respiratory examination, the child was observed to have tachypnea and rhonchi in the right lung fields. The rest of the examination, including systemic examination of the cardiovascular system, was unremarkable.

**Figure 1:**
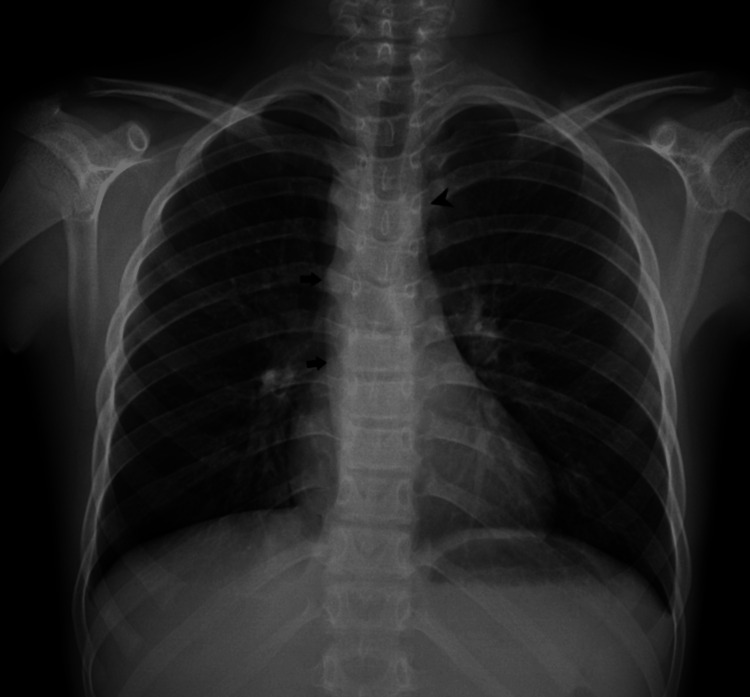
Chest radiograph in the posteroanterior view. Chest radiograph in the posteroanterior view shows the absence of the aortic knuckle on the left side of the mediastinum (black arrowhead) and the shadow of the descending thoracic aorta on the right side (black arrows).

A chest radiograph was obtained to look for lung pathologies, which revealed the absence of an aortic knuckle on the left side of the mediastinum, and the right border of the descending thoracic aorta was noted on the right side of the spine (Figure 1). Based on these findings, a right-sided aortic arch was suspected.

**Figure 2:**
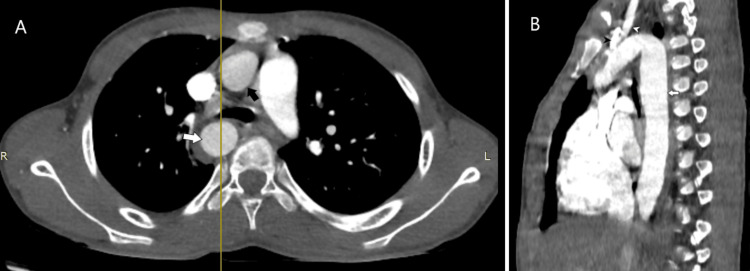
Contrast-enhanced CT thoracic angiography in (A) axial section and (B) sagittal reformatted image. CT: Computed tomography. Contrast-enhanced CT thoracic angiography axial section (A) shows the ascending thoracic aorta (black arrow) and descending thoracic aorta (white arrow) on the right side of the spine. Sagittal reformatted image (B) at the level of the yellow line shows the descending thoracic aorta (white arrow) giving rise to the right subclavian artery (black arrowhead) and right common carotid artery (white arrowhead).

**Figure 3:**
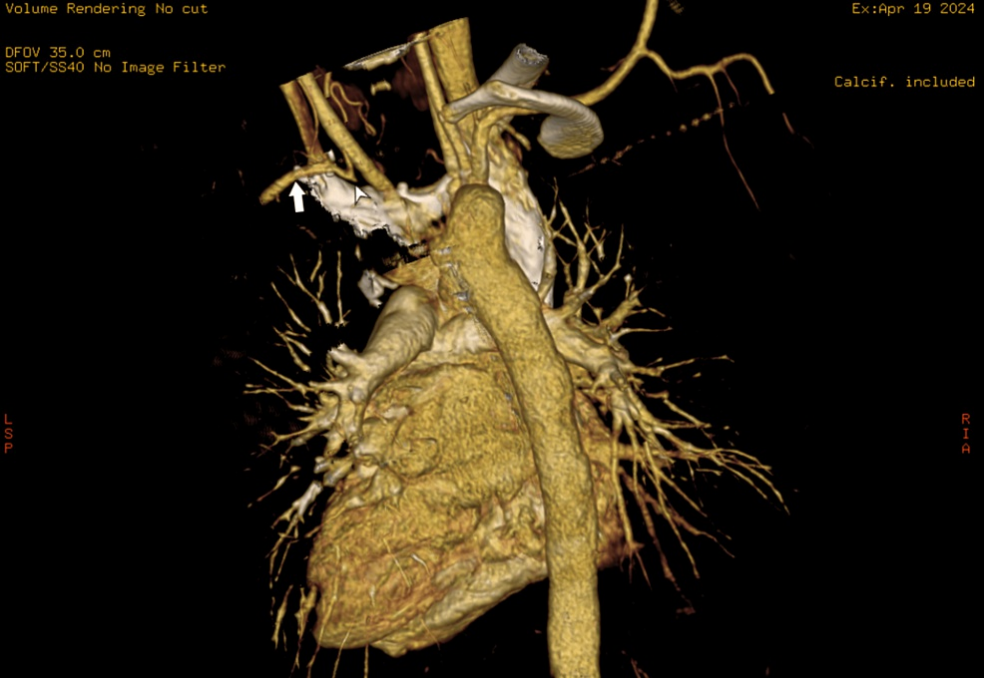
Contrast-enhanced CT thoracic angiogram volume-rendered image. CT: Computed tomography. The contrast-enhanced CT thoracic angiogram volume rendered image shows the left subclavian artery (white arrow) not seen communicating with the aorta and arising from the left vertebral artery (white arrowhead).

**Figure 4:**
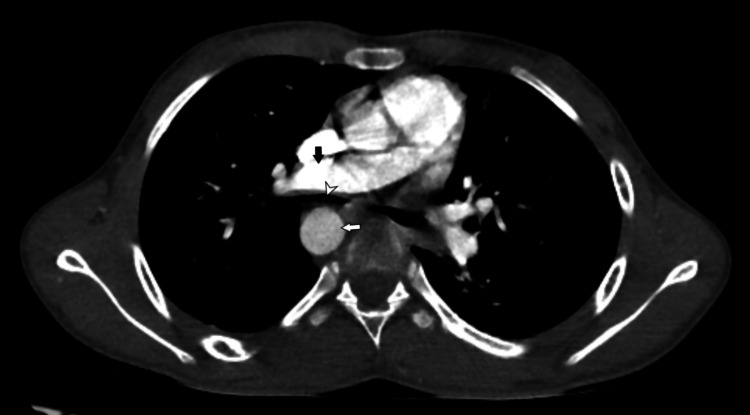
Axial section of contrast-enhanced CT thorax just below the level of the carina. CT: Computed tomography. The right main bronchus (white arrowhead) is compressed between the right main pulmonary artery (black arrow) and the right-sided descending thoracic aorta (white arrow).

**Figure 5:**
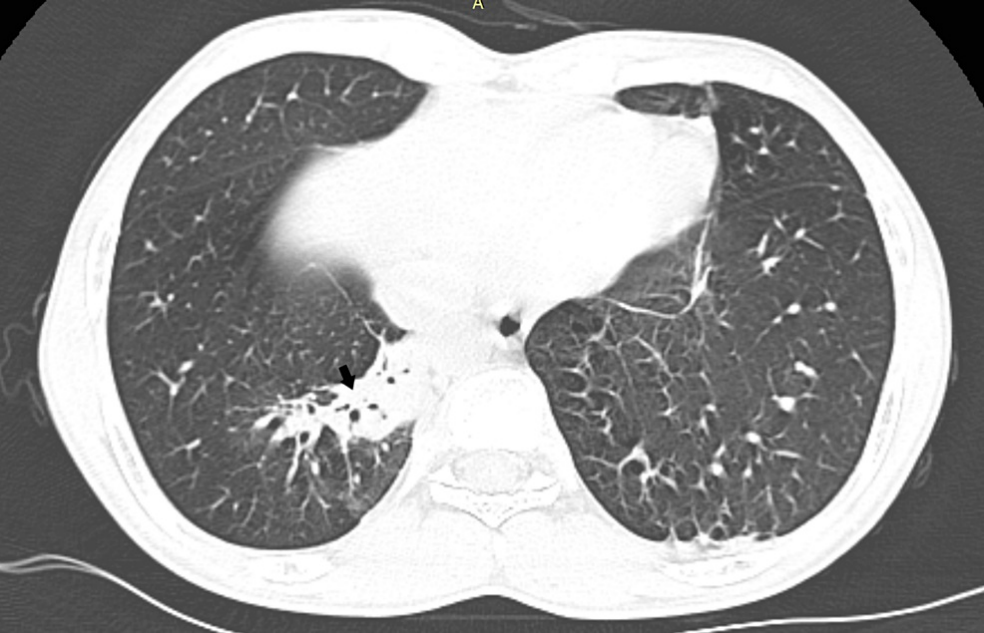
Axial section of high-resolution CT thorax in the lung window. CT: Computed tomography. Patchy opacities with air bronchograms in the medial basal segment of the right lower lobe (black arrow), suggestive of consolidation.

A computed tomography (CT) thoracic angiogram was obtained using the bolus tracking method with the administration of 60 mL of intravenous iohexol contrast followed by a 20 mL saline chase in the region of interest at the root of the aorta to further assess the type and variations of right-sided aortic arch and look for associated complications. On CT angiography, there was a right-sided aortic arch giving three branches: the left common carotid, the right common carotid, and the right subclavian (Figures 2, 3). The left subclavian was not visualized in the mediastinum and was seen to communicate with a small-caliber left vertebral artery at the level of the first thoracic vertebra, with normal contrast opacification noted distal to it. There was no anomalous vessel joining the left subclavian artery to the aorta or the pulmonary artery. A right-sided tracheal bronchus was noted arising just above the carina. The right main bronchus was compressed between the right-sided descending thoracic aorta and the right pulmonary artery, with an anteroposterior diameter of 4mm (Figure 4). Additionally, a patchy area of consolidation was observed in the medial basal segment of the right lower lobe (Figure 5).

**Figure 6:**
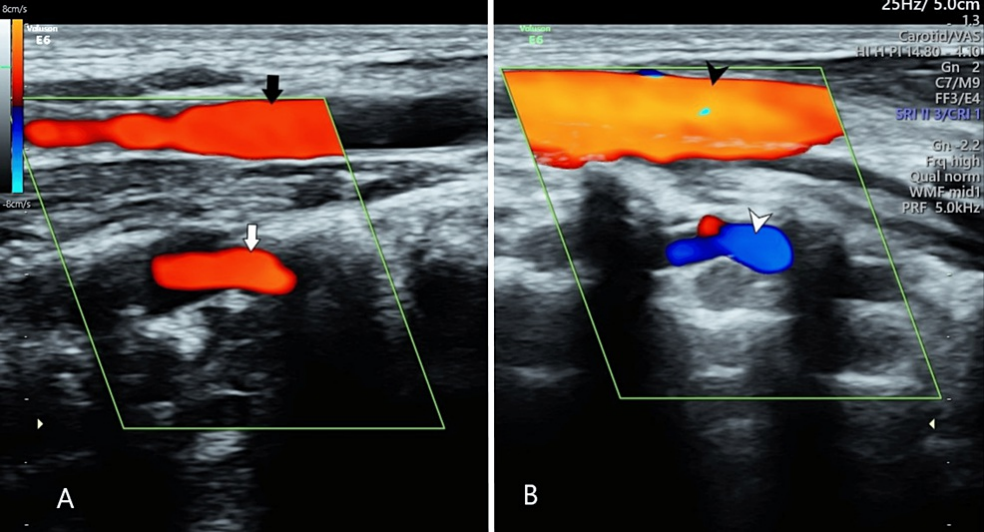
Ultrasound examination of neck vessels. Ultrasound examination of neck vessels in the longitudinal section on the right (A) and left (B) sides shows normal flow direction in the right common carotid artery (black arrow), right vertebral artery (white arrow), and left common carotid artery (black arrowhead) with reversal of flow direction in the left vertebral artery (white arrowhead).

**Figure 7:**
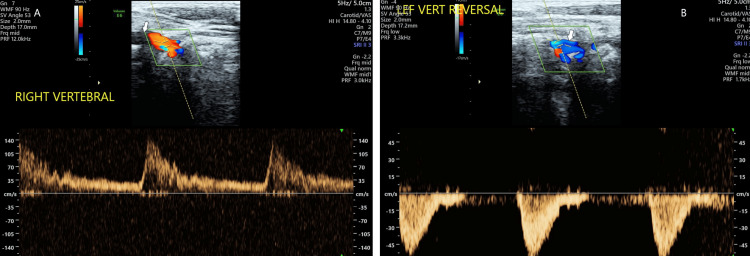
Pulsed wave Doppler examination of bilateral vertebral arteries. Pulsed wave Doppler examination of the right vertebral artery (A) shows low-resistance, high-velocity flow, while the left vertebral artery (B) shows reversed, high-resistance flow.

Ultrasound of the neck was performed using a high-frequency linear probe to assess flow in the vertebral arteries. The right vertebral artery was prominent with high-velocity, low-resistance flow on the Doppler study, whereas the left vertebral artery showed flow reversal with a high-resistance waveform (Figures 6, 7).

**Figure 8:**
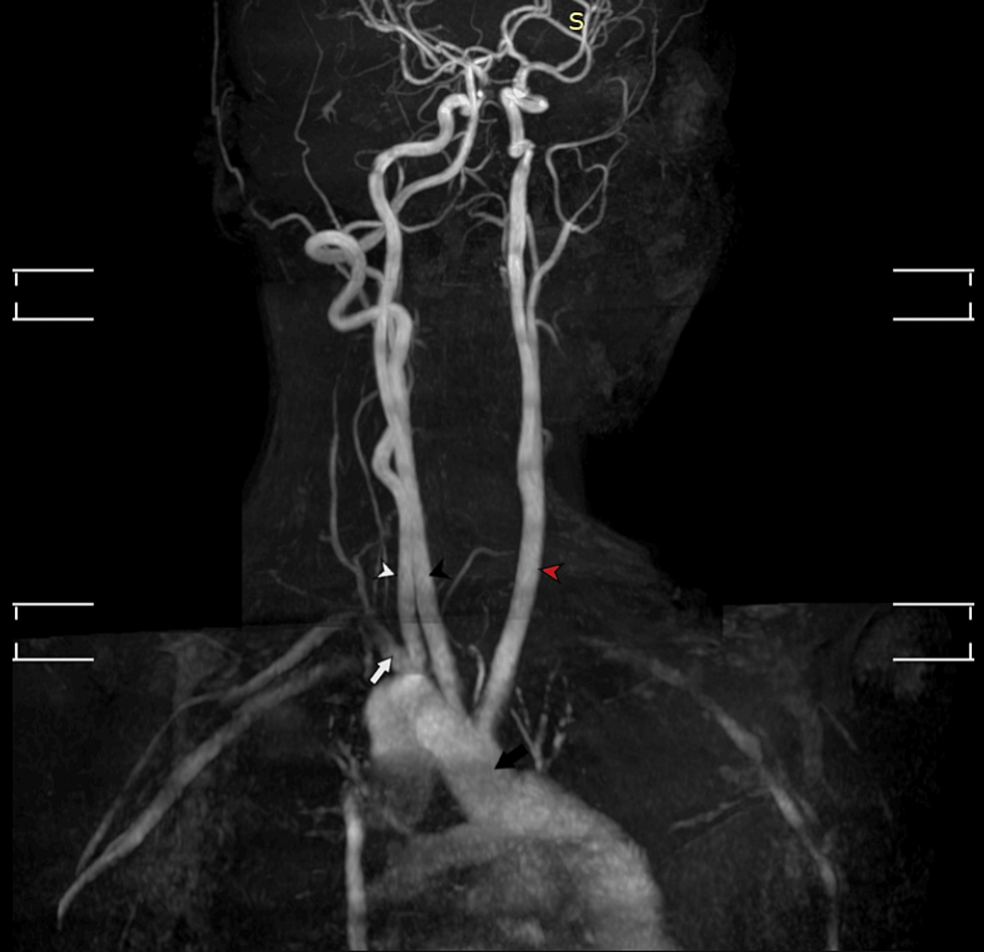
TOF-MRA MOTSA maximum intensity projection image of neck vessels. TOF-MRA: Time-of-flight magnetic resonance angiography; MOTSA: multiple overlapping thin slab acquisition. The right aortic arch (black arrow) gives rise to the left common carotid artery (red arrowhead), right common carotid artery (black arrowhead), and right subclavian artery (white arrow) with the right vertebral artery (white arrowhead) noted arising from the proximal portion of the right subclavian artery. The left vertebral artery and proximal portion of the left subclavian artery could not be visualized.

**Figure 9:**
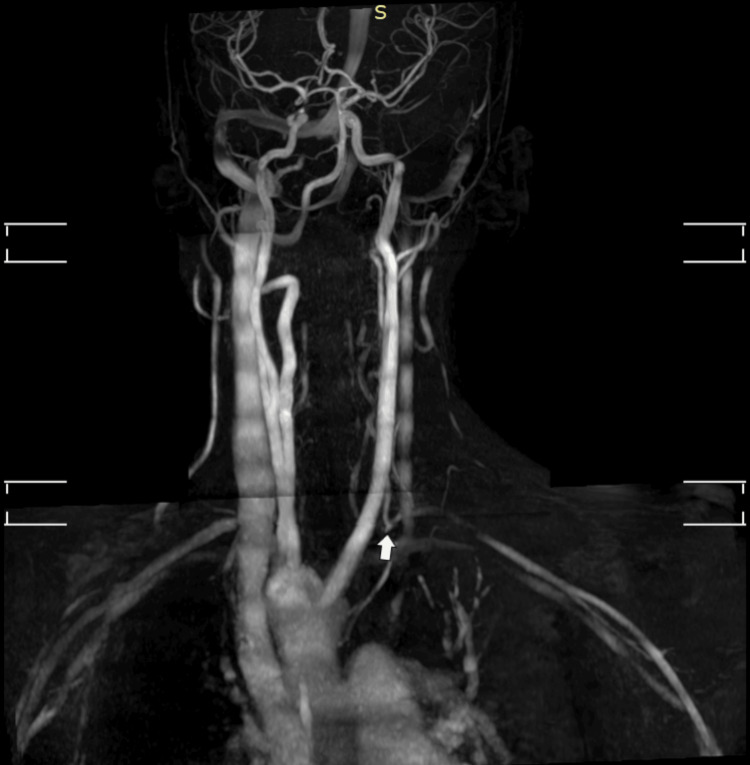
TOF-MRA MOTSA repeated after removing the saturation bands. TOF-MRA: Time-of-flight magnetic resonance angiography; MOTSA: multiple overlapping thin slab acquisition. Maximum intensity projection image showing the left vertebral artery giving rise to the left subclavian artery (white arrow).

Time-of-flight magnetic resonance angiography (TOF-MRA) multiple overlapping thin slab acquisition (MOTSA) of neck vessels was performed, revealing the absence of flow-related enhancement in the left vertebral artery (Figure 8). This was likely due to the reversal of flow in the left vertebral artery, which, because of the saturation bands placed in TOF-MRA to saturate the venous flow, did not produce a signal related to flow in the opposite direction. Upon repeating TOF-MRA with the saturation bands removed, flow enhancement was noted in the left vertebral artery, which was observed joining the left subclavian artery (Figure 9). No flow enhancement was noted in the subclavian artery proximal to the joining of the vertebral artery.

The child underwent further evaluation for associated cardiac anomalies with echocardiography, which yielded unremarkable results.

Considering the above findings, the child was re-evaluated clinically. Upon examination, it was noted that the left radial pulse was feeble compared to the right, and the blood pressure in the left arm was 80/60 mmHg, while it was 110/74 mmHg in the right arm.

Treatment for the lower respiratory tract infection consisted of a three-day course of oral clarithromycin and syrup of chlorpheniramine maleate. The child and their parents were counseled regarding the condition and the potential risks of developing symptoms due to vertebrobasilar insufficiency, such as dizziness, vertigo, syncope, ataxia, sensory disturbances, and dysarthria. Additionally, they were informed about symptoms related to reduced blood flow in the arm, such as weakness, sensory disturbances, tingling, and numbness.

Following the antibiotic course, the child remained clinically stable. Surgical management, including bypass grafting between the left common carotid and left subclavian artery, was recommended. However, since the child did not exhibit severe symptoms at the time, both the child and his parents were not willing to proceed with surgical intervention. The child is currently under close follow-up.

## Discussion

A right-sided aortic arch is an anatomical variation characterized by its course to the right of the trachea. Two types of right-sided aortic arch were originally described by Edwards [3] and Felson and Palayew [4]. Right-sided aortic arch with mirror-image branching was classified as type I and the aberrant left subclavian artery with associated Kommerell’s diverticulum was classified as type II [2]. However, there are variations in the naming of various types in the literature.

Later, in 1970, Shuford et al. described a third type of right aortic arch, which was associated with an isolated left subclavian artery [5]. In this type, the left subclavian and the aorta are no longer connected. This can be explained by the embryologic interruption of the left arch at two levels: the first one between the left common carotid artery and the left subclavian artery, and the second one distal to the attachment of the left ductus. The initial segment of the embryonic left arch transforms into the left common carotid artery, emerging as the foremost branch of the right arch, followed by the right common and right subclavian arteries sequentially [5]. The blood supply to the left subclavian will be from the pulmonary artery from a left ductus [1], which will close after birth due to reduced pulmonary resistance, after which flow can be from the ipsilateral vertebral artery in a retrograde manner or can be from a collateral branch arising from the aorta [6]. This reversal of blood flow in a vertebral artery associated with a reduction in blood supply to the brain is called 'Subclavian steal syndrome' [6].

Clinically, the majority of patients with isolated left subclavian artery are asymptomatic, with few patients presenting with shortness of breath and respiratory infections, and very rarely, they can present with coldness of the skin over the ipsilateral arm. However, later in life, as they develop atherosclerotic changes in the arteries, they can get symptoms of vertebrobasilar insufficiency [6]. With a right aortic arch, the trachea can be compressed by the main pulmonary trunk, aortic arch, and ligamentum arteriosus [7]. This can cause symptoms in children such as wheezing and makes them more prone to develop respiratory infections.

Radiological evaluation of the right-sided aortic arch includes a chest radiograph, which may not provide a diagnosis; however, as seen in our case, it can suggest the possibility of a right-sided aortic arch. A CT thoracic angiogram is the preferred modality to evaluate the type of right-sided aortic arch [1] and can also be used to look for associated variations and complications, such as compression over the bronchus or carina, as seen in our case. Ultrasound Doppler evaluation helps to identify flow reversal in the vertebral artery, which can diagnose subclavian steal syndrome.

The right-sided aortic arch is also known to be associated with other congenital cardiac and extracardiac anomalies, with cyanotic congenital heart disease, especially tetralogy of Fallot and truncus arteriosus being common [1], and also a few genetic syndromes, such as 22q11.2 deletion (DiGeorge syndrome) [8]. However, in our case, echocardiography was normal, and no signs of 22q11.2 deletion, such as thymic hypoplasia, were noted.

A similar case was reported by Ahmed et al. in a 47-year-old male with a right aortic arch with left subclavian steal syndrome who had presented with symptoms of headaches, dizziness, blurry vision, and hearing loss in his left ear. They reported that most of the patients develop symptoms of vertebrobasilar insufficiency in the fourth or fifth decade of life due to a lack of compensatory mechanisms [9]. The usual recommended treatment for isolated left subclavian artery with subclavian steal syndrome is a bypass graft between the left common carotid artery and left subclavian artery [10], which can help improve the symptoms of vertebrobasilar insufficiency.

## Conclusions

In conclusion, when evaluating a case of suspected right aortic arch, it is essential to conduct a comprehensive diagnostic workup. A chest radiograph should be obtained to confirm the presence of a right aortic arch and assess secondary lung pathologies. A contrast-enhanced CT thoracic angiogram is valuable for delineating the aortic branching pattern, classifying the type of right aortic arch, and detecting potential complications such as aberrant left subclavian artery or tracheal compression. Additionally, CT can identify secondary lung pathologies like consolidations or ground-glass opacities.

TOF-MRA of neck vessels provides detailed visualization of vessel anatomy, but proper planning is crucial to avoid non-visualization of the left vertebral artery due to retrograde flow. This can be mitigated by removing saturation bands during MRA planning or using contrast-enhanced MRA. Echocardiography should be performed to rule out associated cardiac anomalies. Vascular bypass graft is the treatment of choice for an isolated left subclavian artery with subclavian steal syndrome, which can alleviate symptoms of vertebrobasilar insufficiency and improve patient outcomes.
